# Supplementary material for: Key Stakeholders’ Perspectives on Implementation and Scale up of HIV Self-Testing in Rwanda
Source: Diagnostics (Basel). 2020 Apr 1;10(4):194. doi: 10.3390/diagnostics10040194 (PMC7235833; doi:10.3390/diagnostics10040194)
Supplement: Supplementary file 1 [file diagnostics-10-00194-s001.zip › Supplemental File 2.docx]

**Supplemental File 2**: Detailed analysis of the interview transcripts against the Consolidated Framework for Implementation Research

| **Consolidated Framework for Implementation Research Domains and Constructs** | **Findings from key stakeholders’ responses** |
| --- | --- |
| **I. INTERVENTION CHARACTERISTICS** |  |
| **A Intervention Source** Perception of key stakeholders about whether the intervention is externally or internally developed. | Stakeholders perceive that the intervention is developed from the external entity e.g. WHO. Although Rwanda internal entities e.g. MoH/RBC also played a critical role. |
| **B Evidence Strength & Quality** Stakeholders’ perceptions of the quality and validity of evidence supporting the belief that the intervention will have desired outcomes. | Currently, there are guidelines in place to direct HIVST. Before kits are even distributed, validation is performed by the National Reference Laboratory. This ensure that quality kits are received and for this, only rare cases of false results have been reported. Among others, a pilot study was done on a few pharmacies and other healthcare facilities in Kigali, which yielded fruitful results. Stakeholders therefore perceive this is a good initiative to reach hard to reach populations. |
| **C Relative advantage** Stakeholders’ perception of the advantage of implementing the intervention versus an alternative solution | Even with conventional HIV testing services, most stakeholders have a general impression that there is still a proportion of the population that does not utilize these services. Possible reasons could be related to stigma, time constraints and confidentiality issues. Implementing this intervention would mean that this gap will be bridged. |
| **D Adaptability** The degree to which an intervention can be adapted, tailored, refined, or reinvented to meet local needs | Throughout pilot testing and feedback mechanisms, the nature of this intervention demonstrated that there could be room for refining the method to suit local needs. Issues related to counselling for instance calls for incorporation of hotlines like a number of stakeholders suggested. Cost of the kits as well, should be reduced according to most stakeholders by means of subsidies to cater for everyone. The ultimate goal is to have a method that meets local needs. |
| **E Trialability** The ability to test the intervention on a small scale in the organization [8], and to be able to reverse course (undo implementation) if warranted. | The intervention under study was initially rolled out on a small scale in pilot phases. Only a few pharmacies and health care facilities in Kigali were involved at first. This allowed key players in the implementation process to identify rooms for improvement. It is for that purpose that evaluation and reflection should be done throughout all stages. Training needs for instance and lack of awareness in regards to HIVST kits have been pointed out by most stakeholders as challenges which calls for Rwanda governments’ attention before rolling out the intervention on a large scale. |
| **F Complexity** Perceived difficulty of implementation, reflected by duration, scope, radicalness, disruptiveness, centrality, and intricacy and number of steps required to implement | Not assessed |
| **G Design Quality and Packaging** Perceived excellence in how the intervention is bundled, presented, and assembled | The intervention has been presented well to a certain extent, however, most believe it could have been approached differently. Different areas should have unfolded well given that proper strategies were in place. A good example some stakeholders gave is inadequate involvement of some key implementation partners for starters and poor planning in sensitization of the population towards HIVST. |
| **H Cost** Costs of the intervention and costs associated with implementing that intervention including investment, supply, and opportunity costs. | The government of Rwanda invested resources in a various areas including training of pharmacies, healthcare facilities, purchasing kits from vendors and availing them for free, piloting projects and putting guidelines in place. These all gave proof of the government investment. Overall, HIVST implementation is the price the government had to pay, not to miss out on the opportunity of reaching out to key populations. |
| **II. OUTER SETTING** |  |
| **A Patient Needs & Resources** The extent to which patient needs, as well as barriers and facilitators to meet those needs are accurately known and prioritized by the organization. | Patients’ welfare are the ultimate goal for this intervention. The fact that this method is tailored to cater for key populations such as victimized groups including sex workers speaks volume in itself. The idea is to link everyone to treatment and care. Although there has been concerns in terms of related issues e.g., testing without counselling, or cohesion, these issues are well thought out through to meet patients’ needs as the transcripts demonstrate. The intervention is tailored to answer to questions such as what do patients want. What is the best approach to deliver this services that patients are comfortable with? One stakeholder for example reported that since men hardly come to health facilities, kits are rather given to their wives who turn up. |
| **B Cosmopolitanism** The degree to which an organization is networked with other external organizations. | Various partners played a critical role in this intervention. These include the government of Rwanda, Ministry of Health, PSF, RBC, AHF and CDC. The goal is to implement HIVST and ultimately increase the uptake of HIV testing in the country at large. These partners are collaborating together in a combined effort to achieve fruitful end results. However, some stakeholders believed that there were challenges in adequately involving some key implementation partners which in the end derailed the implementation process. |
| **C Peer Pressure** Mimetic or competitive pressure to implement an intervention; typically because most or other key peer or competing organizations have already implemented or in a bid for a competitive edge. | This intervention is fueled by the WHO recommendations to roll out HIVST. Other driving forces could be research that demonstrated that the significant proportion of the population still did not know their HIV statuses. With these and some political intent, the government took initiative. |
| **D External Policy & Incentives** A broad construct that includes external strategies to spread interventions including policy and regulations (governmental or other central entity), external mandates, recommendations and guidelines, pay-for-performance, collaboratives, and public or benchmark reporting. | WHO guidelines recommended HIVST roll out on a global scale, however, other alterities were not accessed in the manuscript. |
| **III. INNER SETTING** |  |
| **A Structural Characteristics** The social architecture, age, maturity, and size of an organization. | Not assessed |
| **B Networks & Communications** The nature and quality of webs of social networks and the nature and quality of formal and informal communications within an organization. | The government of Rwanda gave the MoH the mandate to roll out HIVST. Although networks and communications between partners remained vivid, certainly there were a few communication barriers. Some stakeholders for instance didn’t receive feedback on pilot project that took place and were generally not on the same pace in terms of information dissemination. |
| **C Culture** Norms, values, and basic assumptions of a given organization. | RBC has clear objectives that are well communicated to all partners/stakeholders. This is to implement HIVST, an intervention that will result in increased uptake of HIV testing. Most values are centered around patients’ safety, and ethics that governs all medical research when human subjects are involved e.g. confidentiality and respect of human dignity. |
| **D Implementation Climate** The absorptive capacity for change, shared receptivity of involved individuals to an intervention and the extent to which use of that intervention will be rewarded, supported, and expected within their organization | On account that the government of Rwanda has been committed to continual improvement in HIV testing, it is notable that various measure have been put into place to ensure more people get tested. Stakeholders have therefore been properly involved if not all from planning stages to implementation. Proper integration of HIV programs into the healthcare system have also been effective. The common goal for implementation have thus been supported by all stakeholders. |
| **D**.**1 Tension for Change** The degree to which stakeholders perceive the current situation as intolerable or needing change. | A number of stakeholders reported a gap with the conventional HIV testing services. The main highlighted was not being able to reach certain key populations. This is said to have been responsible for not reaching the UNAIDS first 90 target. Most stakeholders were of the opinion that this gap among others has been the tension for change. |
| **D.2 Compatibility** The degree of tangible fit between meaning and values attached to the intervention by involved individuals, how those align with individuals’ own norms, values, and perceived risks and needs, and how the intervention fits with existing workflows and systems. | All the stakeholders are familiar with conventional HIV testing services being unable to cater for all, it is a fact. There has also been a growing need to include the entire population in the fight against the epidemic. All stakeholders had from a medical/ health background and for that, they have an understanding that efforts towards HIVST is in the right direction. The only emphasis is that this intervention is approached with delicacy. Challenges such as Pre and post counselling or high costs of the kits should be ironed out prior. |
| **D.3 Relative Priority** Individuals’ shared perception of the importance of the implementation within the organization. | The majority of stakeholders are under the general impression that HIVST strategy is for a good course. They also believe that taking this step is paramount; as it is the passageway to link individuals to treatment and care. |
| **D.**4 **Organizational Incentives & Rewards** Extrinsic incentives such as goal-sharing awards, performance reviews, promotions, and raises in salary and less tangible incentives such as increased stature or respect. | Not assessed |
| **D.5 Goals and Feedbac**k The degree to which goals are clearly communicated, acted upon, and fed back to staff and alignment of that feedback with goals. | Not assessed |
| **D.6 Learning Climate** A climate in which: a) leaders express their own fallibility and need for team members’ assistance, and input; b) team members feel that they are essential, valued, and knowledgeable partners in the change process; c) individuals feel psychologically safe to try new methods; and d) there is sufficient time and space for reflective thinking and evaluation. | Not assessed. |
| **E Readiness for Implementation** Tangible and immediate indicators of organizational commitment to its decision to implement an intervention | Factors that demonstrated readiness for implementation include training that took effect, knowledge sharing e.g. through mobilization and investing resources such labor in terms of the team that led implementation and capital to procure kits if not all. |
| **E.1 Leadership** Engagement Commitment, involvement, and accountability of leaders and managers with the implementation. | Stakeholders perceive that leaders have been committed. This is demonstrated by the views on the urge to change different procedures for a greater impact. Many suggested that the government should come up with strategies that’ll attract the public to come onboard. |
| **E.2 Available Resources** The level of resources dedicated for implementation and on-going operations including money, training, and education, physical space, and time. | Stakeholders highlighted training models that have been going on. So much time has been dedicated to the project already by show of efforts and other resources invested including human capital. |
| **E.3 Access to knowledge and information** Ease of access to digestible information and knowledge about the intervention and how to incorporate it into work tasks. | The majority of the stakeholders’ responses demonstrated easy access to digestible knowledge, most shared the same perception of the same language in terms of the intervention. |
| **IV. CHARACTERISTICS OF INDIVIDUALS** |  |
| **A Knowledge & Beliefs about the Intervention**, Individuals’ attitudes toward and value placed on the intervention as well as familiarity with facts, truths, and principles related to the intervention. | As different stakeholders were interviewed, all responses demonstrated enthusiasm towards implementing the intervention. Even the ones who were bit concerned about a number of challenges offered possible solutions. Also, familiarity with sound truths, facts and skills were demonstrated towards HIVST. |
| B Self-efficacy Individual belief in their own capabilities to execute courses of action to achieve implementation goals | Stakeholders demonstrated confidence in what they were doing and they also had an idea of the entire implementation process. The majority believed that their efforts contributes greatly to implementation success. |
| **C Individual Stage of Change** Characterization of the phase an individual is in, as he or she progresses toward skilled, enthusiastic, and sustained use of the intervention. | Throughout in-depth interviews, all stakeholders demonstrated knowledge of facts in regards to HIVST. They believed that if the intervention is approached in a way that irons out a few challenges, there will be definite success. Everyone answered yes when asked if they thought the intervention could work. |
| **D Individual Identification with Organization** A broad construct related to how individuals perceive the organization and their relationship and degree of commitment with that organization. | Stakeholders were of the viewpoint that the government of Rwanda is highly committed and loyal in bringing forth the implementation, many also suggested different ways on how the government should approach the intervention for high turnover. |
| **E Other Personal Attributes** A broad construct to include other personal traits such as tolerance of ambiguity, intellectual ability, motivation, values, competence, capacity, and learning style. | Not assessed |
| **V. PROCESS** |  |
| A Planning The degree to which a scheme or method of behavior and tasks for implementing an intervention are developed in advance and the quality of those schemes or methods. | Apart from involving stakeholders and partners, a lot went in the planning phase. This entails drawing up policies and guidelines to regulate the intervention. Strategies for kits regulation were in place as well as to whom will get training and when. The pilot project that placed the implementation to a test for instance was planned prior and which areas were more people will be reached. Moreover, quality assurances of kits being used and accreditation of all centers disseminating the kits among others all form part of the planning. |
| **B Engaging** Attracting and involving appropriate individuals in the implementation and use of the intervention through a combined strategy of social marketing, education, role modeling, training, and other similar activities. | Various activities have been in place to attract/sensitize the public on the new intervention e.g. different mobilizations, adverts from pharmacies, campaigns, and formal announcements in terms of policies that have been drafted. All these made an impact to lure individuals. Most stakeholders however believed that a lot more could have been done. |
| **B.1 Opinion Leaders** Individuals in an organization who have formal or informal influence on the attitudes and beliefs of their colleagues with respect to implementing the intervention | Most key players who have been selected to head the implementation process includes influential people who have the capacity to influence the population through authority, status and expertise. Stakeholders demonstrated that they were there to influence change, the government in particular. |
| **B.2 Formally appointed internal implementation leaders** Individuals from within the organization who have been formally appointed with responsibility for implementing an intervention as coordinator, project manager, team leader, or other similar role. | Various appointed stakeholders similarly are in positions that influence people, head of centers for RBC for instance have been given as an example. With this approach, people on the ground are more receptive of the approach e.g. training. |
| **B.3 Champions** “Individuals who dedicate themselves to supporting, marketing, and ‘driving through’ an [implementation], overcoming indifference or resistance that the intervention may provoke in an organization. | Not assessed |
| **B.4 External Change Agents** Individuals who are affiliated with an outside entity who formally influence or facilitate intervention decisions in a desirable direction. | Stakeholders pointed to the WHO guidelines. Researchers interviewed brought forth current statistics of HIV status in the country. |
| **C Executing** Carrying out or accomplishing the implementation according to plan. | Stakeholders outlined different challenges to the implementation process and proposed strategies to address these. |
| **D Reflecting & Evaluating** Quantitative and qualitative feedback about the progress and quality of implementation accompanied with regular personal and team debriefing about progress and experience. | Not assessed |
