# Supplementary material for: Key Stakeholders’ Perspectives on Implementation and Scale up of HIV Self-Testing in Rwanda
Source: Diagnostics (Basel). 2020 Apr 1;10(4):194. doi: 10.3390/diagnostics10040194 (PMC7235833; doi:10.3390/diagnostics10040194)
Supplement: Supplementary file 1 [file diagnostics-10-00194-s001.zip › Supplementary File 1 Interview Guide.docx]

**Key stakeholders’ perspectives on implementation and scale up of HIV self-testing in Rwanda**

Supplementary File 1: Interview Guide

| **Ministry of Health/ Rwanda Biomedical Center and NGO stakeholders in-depth interview**  Question | Remarks |
| --- | --- |
| Introduction: “Hello, my name is XXX. I will conduct the interview, record and take notes. I invited you to discuss HIV self-testing and your perception on implementation of HIV self- testing in Rwanda. I will ask you several open questions. Your personal opinions and views are very important for us. There are no right or wrong answers. Please feel welcome to express yourself freely during the interview.  This conversation will be recorded on tape. This is only for purpose of the research, only the lead researcher indicated on the consent form (and I) will listen to the tape. No names or personal information will be used in the report.  The interview will last for about forty minutes. Is everything clear about the course of the interview? | Before starting the interview all participants will be informed about the purpose of the discussion, confidentiality and practical issues. |
| 1. Please tell me what you know about HIV self-testing in Rwanda | For acquaintance with the participants and gradually focusing the discussion on the study topic |
| 2. Are you involved in HIV self-testing implementation in Rwanda?  *If YES what is your current role? If NO what will be your role.* | To probe information on participant’s role in implementation of HIVST in Rwanda. |
| 3. What is your perception toward current HIV testing services in Rwanda? What has worked? What still needs to be strengthened?  *Current HIV testing services structure in Rwanda at different levels*  *Perceived challenges with current HIV testing services in Rwanda*  *Perceptions on system readiness for HIVST implementation in Rwanda*  *Perception on how HIVST will complement current HIV testing services* | Introduction question aims to explore participant perspective on current HIV testing services.  The sub-questions to the introduction question will be aimed to assess perceptions toward current organizational characteristics, culture, implementation climate and readiness |
| 4. Given your experience with HIV testing serves in Rwanda, what do you think will need to be set up before HIV self-testing can be rolled up at large scale successfully?  *Adaptability of HIVST in intervention in Rwanda*  *Complexity of HIVST implementation in Rwanda*  *Resources currently available for HIVST implementation in Rwanda and perceived shortfalls*  *Perceptions on costs associated with HIVST implementation in Rwanda for government and for the users* | Introduction question aims to explore participant perspective on adaptability of HIVST in Rwanda.  The sub-questions to the introduction question will be aimed to assess perceptions toward complexity, resources and cost of HIVST implementation. |
| 5. What are your perceptions on the existing HIV testing policies and what regulatory challenges do you perceive for HIVST policy? | To assess participant perception on the existing HIV testing policies and perceived regulatory challenges with HIVST policy.  The sub-questions to the introduction question will aim to assess more information on stated potential challenges. |
| 6. What are your general impressions on HIV self-testing? | To explore positive and negative perceptions toward HIV self-testing.  Sub- questions will be used to explore the positive and negative perceptions toward HIV self-testing based on the participant response. |
| 7. In conclusion, what is your recommendation on how Ministry of Health should implement HIV self-testing in Rwanda to ensure uptake and increase use of HIV testing services? | This question will be used to further explore any additional perceptions on implementation. Recommendations will be explored from the participants on what they feel would make HIVST implantation a success in Rwanda.  At the end of the interview, the participants will be given the opportunity to add remarks or suggestions related to the subject topic. |

| **Health care provider (HIV Clinic nurse/ counsellor) in-depth interview**  Question | Remarks |
| --- | --- |
| Introduction: “Hello, my name is XXX. I will conduct the interview, record and take notes. I invited you to discuss HIV self-testing and your perception on implementation of HIV self- testing in Rwanda. I will ask you several open questions. Your personal opinions and views are very important for us. There are no right or wrong answers. Please feel welcome to express yourself freely during the interview.  This conversation will be recorded on tape. This is only for purpose of the research, only the lead researcher indicated on the consent form (and I) will listen to the tape. No names or personal information will be used in the report.  The interview will last for about forty minutes. Is everything clear about the course of the interview? | Before starting the interview all participants will be informed about the purpose of the discussion, confidentiality and practical issues. |
| 1. Please tell me what you know about HIV self-testing in Rwanda | For acquaintance with the participants and gradually focusing the discussion on the study topic |
| 2. Are you involved in HIV self-testing implementation in Rwanda?  *If YES what is your current role? If NO what will be your role.* | To probe information on participant’s role in implementation of HIVST in Rwanda. |
| 3. What is your perception toward current HIV testing services in Rwanda? What has worked? What still needs to be strengthened?  *Current HIV testing services structure in Rwanda at different levels*  *Perceived challenges with current HIV testing services in Rwanda*  *Perceptions on system readiness for HIVST implementation in Rwanda*  *Perception on how HIVST will complement current HIV testing services* | Introduction question aims to explore participant perspective on current HIV testing services.  The sub-questions to the introduction question will be aimed to assess perceptions toward current organizational characteristics, culture, implementation climate and readiness |
| 4. Given your experience with HIV testing serves in Rwanda, what do you think will need to be set up before HIV self-testing can be rolled up at large scale successfully?  *Adaptability of HIVST in intervention in Rwanda*  *Complexity of HIVST implementation in Rwanda*  *Resources currently available for HIVST implementation in Rwanda and perceived shortfalls*  *Perceptions on costs associated with HIVST implementation in Rwanda for government and for the users* | Introduction question aims to explore participant perspective on adaptability of HIVST in Rwanda.  The sub-questions to the introduction question will be aimed to assess perceptions toward complexity, resources and cost of HIVST implementation. |
| 5. What are your perceptions on the existing HIV testing policies and what regulatory challenges do you perceive for HIVST policy? | To assess participant perception on the existing HIV testing policies and perceived regulatory challenges with HIVST policy.  The sub-questions to the introduction question will aim to assess more information on stated potential challenges. |
| 6. What are your general impressions on HIV self-testing? | To explore positive and negative perceptions toward HIV self-testing.  Sub- questions will be used to explore the positive and negative perceptions toward HIV self-testing based on the participant response. |
| 7. What are the anticipated challenges with HIV self-testing uptake among men in your district? | To assess health care provider perceived barriers to uptake of HIVST by men |
| 8. In conclusion, what is your recommendation on how Ministry of Health should implement HIV self-testing in Rwanda to ensure uptake and increase use of HIV testing services? | This question will be used to further explore any additional perceptions on implementation. Recommendations will be explored from the participants on what they feel would make HIVST implantation a success in Rwanda.  At the end of the interview, the participants will be given the opportunity to add remarks or suggestions related to the subject topic. |

| **National Reference Laboratory staff in-depth interview**  Question | Remarks |
| --- | --- |
| Introduction: “Hello, my name is XXX. I will conduct the interview, record and take notes. I invited you to discuss HIV self-testing and your perception on implementation of HIV self- testing in Rwanda. I will ask you several open questions. Your personal opinions and views are very important for us. There are no right or wrong answers. Please feel welcome to express yourself freely during the interview.  This conversation will be recorded on tape. This is only for purpose of the research, only the lead researcher indicated on the consent form (and I) will listen to the tape. No names or personal information will be used in the report.  The interview will last for about forty minutes. Is everything clear about the course of the interview? | Before starting the interview all participants will be informed about the purpose of the discussion, confidentiality and practical issues. |
| 1. Please tell me what you know about HIV self-testing in Rwanda | For acquaintance with the participants and gradually focusing the discussion on the study topic |
| 2. Are you involved in HIV self-testing implementation in Rwanda?  *If YES what is your current role? If NO what will be your role.* | To probe information on participant’s role in implementation of HIVST in Rwanda. |
| 3. What is your perception toward current HIV testing services in Rwanda? What has worked? What still needs to be strengthened?  *Current HIV testing services structure in Rwanda at different levels*  *Perceived challenges with current HIV testing services in Rwanda*  *Perceptions on system readiness for HIVST implementation in Rwanda*  *Perception on how HIVST will complement current HIV testing services* | Introduction question aims to explore participant perspective on current HIV testing services.  The sub-questions to the introduction question will be aimed to assess perceptions toward current organizational characteristics, culture, implementation climate and readiness |
| 4. Given your experience with HIV testing serves in Rwanda, what do you think will need to be set up before HIV self-testing can be rolled up at large scale successfully?  *Adaptability of HIVST in intervention in Rwanda*  *Complexity of HIVST implementation in Rwanda*  *Resources currently available for HIVST implementation in Rwanda and perceived shortfalls*  *Perceptions on costs associated with HIVST implementation in Rwanda for government and for the users* | Introduction question aims to explore participant perspective on adaptability of HIVST in Rwanda.  The sub-questions to the introduction question will be aimed to assess perceptions toward complexity, resources and cost of HIVST implementation. |
| 5. What are your perceptions on the existing HIV testing policies and what regulatory challenges do you perceive for HIVST policy? | To assess participant perception on the existing HIV testing policies and perceived regulatory challenges with HIVST policy.  The sub-questions to the introduction question will aim to assess more information on stated potential challenges. |
| 6. What are your general impressions on HIV self-testing? | To explore positive and negative perceptions toward HIV self-testing.  Sub- questions will be used to explore the positive and negative perceptions toward HIV self-testing based on the participant response. |
| 7. What are the anticipated challenges with HIV self-testing from the laboratory perspective? | To assess laboratory perceived challenges to implementation of HIVST |
| 8. In conclusion, what is your recommendation on how Ministry of Health should implement HIV self-testing in Rwanda to ensure uptake and increase use of HIV testing services? | This question will be used to further explore any additional perceptions on implementation. Recommendations will be explored from the participants on what they feel would make HIVST implantation a success in Rwanda.  At the end of the interview, the participants will be given the opportunity to add remarks or suggestions related to the subject topic. |

| **RBC/Medical and Pharmaceutical Products Department (supply chain) staff in-depth interview**  Question | Remarks |
| --- | --- |
| Introduction: “Hello, my name is XXX. I will conduct the interview, record and take notes. I invited you to discuss HIV self-testing and your perception on implementation of HIV self- testing in Rwanda. I will ask you several open questions. Your personal opinions and views are very important for us. There are no right or wrong answers. Please feel welcome to express yourself freely during the interview.  This conversation will be recorded on tape. This is only for purpose of the research, only the lead researcher indicated on the consent form (and I) will listen to the tape. No names or personal information will be used in the report.  The interview will last for about forty minutes. Is everything clear about the course of the interview? | Before starting the interview all participants will be informed about the purpose of the discussion, confidentiality and practical issues. |
| 1. Please tell me what you know about HIV self-testing in Rwanda | For acquaintance with the participants and gradually focusing the discussion on the study topic |
| 2. Are you involved in HIV self-testing implementation in Rwanda?  *If YES what is your current role? If NO what will be your role.* | To probe information on participant’s role in implementation of HIVST in Rwanda. |
| 3. What is your perception toward current HIV testing services in Rwanda? What has worked? What still needs to be strengthened?  *Current HIV testing services structure in Rwanda at different levels*  *Perceived challenges with current HIV testing services in Rwanda*  *Perceptions on system readiness for HIVST implementation in Rwanda*  *Perception on how HIVST will complement current HIV testing services* | Introduction question aims to explore participant perspective on current HIV testing services.  The sub-questions to the introduction question will be aimed to assess perceptions toward current organizational characteristics, culture, implementation climate and readiness |
| 4. Given your experience with HIV testing serves in Rwanda, what do you think will need to be set up before HIV self-testing can be rolled up at large scale successfully?  *Adaptability of HIVST in intervention in Rwanda*  *Complexity of HIVST implementation in Rwanda*  *Resources currently available for HIVST implementation in Rwanda and perceived shortfalls*  *Perceptions on costs associated with HIVST implementation in Rwanda for government and for the users* | Introduction question aims to explore participant perspective on adaptability of HIVST in Rwanda.  The sub-questions to the introduction question will be aimed to assess perceptions toward complexity, resources and cost of HIVST implementation. |
| 5. What are your perceptions on the existing HIV testing policies and what regulatory challenges do you perceive for HIVST policy? | To assess participant perception on the existing HIV testing policies and perceived regulatory challenges with HIVST policy.  The sub-questions to the introduction question will aim to assess more information on stated potential challenges. |
| 6. What are your general impressions on HIV self-testing? | To explore positive and negative perceptions toward HIV self-testing.  Sub- questions will be used to explore the positive and negative perceptions toward HIV self-testing based on the participant response. |
| 7. What measure need to be put in place prior to implementation of HIV self-testing from the supply chain perspective? | To assess supply chain manager perception on measures to be put in place prior to implementation of HIVST. |
| 8. What are the anticipated challenges with implementation of HIV self-testing from the supply chain perspective? | To assess supply chain manager perceived challenges to implementation of HIVST |
| 9. In conclusion, what is your recommendation on how Ministry of Health should implement HIV self-testing in Rwanda to ensure uptake and increase use of HIV testing services? | This question will be used to further explore any additional perceptions on implementation. Recommendations will be explored from the participants on what they feel would make HIVST implantation a success in Rwanda.  At the end of the interview, the participants will be given the opportunity to add remarks or suggestions related to the subject topic. |
